# Metacognition in nonhuman primates: a review of current knowledge

**DOI:** 10.1007/s10329-024-01169-x

**Published:** 2024-12-14

**Authors:** Lorraine Subias, Noriko Katsu, Kazunori Yamada

**Affiliations:** Graduate School of Human Sciences, Osaka University, 1-2 Yamadaoka, Suita, Osaka Japan

**Keywords:** Metacognition, Nonhuman primates, Phylogenetic comparison, Literature review

## Abstract

Metacognition, the ability to monitor and control one’s own cognitive processes, has long been considered a hallmark of human cognition. However, two decades of research have provided compelling evidence of metacognitive-like abilities in some nonhuman primates. This review synthesizes current knowledge on the subject, highlighting key experimental paradigms and empirical findings, with an emphasis on the latest studies. Thanks to advances in methods and efforts to counter alternative explanations, there is now a consensus that great apes and some macaque species can monitor and control some of their cognitive processes. Despite numerous investigations, however, whether capuchin monkeys are metacognitive remains unclear. Critical gaps persist in our understanding of metacognition across species. We discuss the importance of expanding research to include a wider range of primate species and the potential role of ecological factors in shaping metacognitive capacities. In addition, we consider some promising avenues for future research, including neurophysiological approaches, studies of metacognitive errors, and field experiments.

## Introduction

John H. Flavell introduced the term “metacognition” to describe higher level cognition, or as it is often summarized: “cognition about cognition” (Flavell 1979). Although metacognition has been studied since the early twentieth century, its definition and what distinguishes a cognitive from a metacognitive process are still debated. Some definitions equate it to the higher order thinking skills which allow an agent to monitor and control its mental states, and to form strategies to improve learning and problem-solving (Nelson 1990). Metacognition can take many forms, such as reflecting on one’s way of thinking, knowing what one knows or feels (or not), or even the common tip-of-the-tongue experience. In humans, it relates to feelings of confidence and doubt and the ability to comment on those feelings.

Whether metacognition necessarily involves conscious, awareness is debatable. Researchers have distinguished between these two views in the following manner:

a) *Declarative (also called conceptual) metacognition*, which sees metacognition as a full-fledged mindreading ability, that is, the ability to attribute mental states to oneself and to others in a language-based manner. In this context, metacognition is considered a higher level cognitive ability, specifically associated with epistemic self-awareness and the ability to form metarepresentations of one’s knowledge and beliefs (Carruthers 1989, 2008; Perner and Dienes 2003).

b) *Procedural metacognition*, which do not require conceptual (i.e., linguistically structured mental contents) but rather non-conceptual forms of metacognition resulting in epistemic feelings, such as “feelings of knowing,” “feelings of uncertainty,” and “feelings of confidence” (Koriat 2000; Proust 2006, 2019). Based on this, discussions have centered on whether animals who lack declarative metacognition could possess procedural metacognition, and whether procedural metacognition should be considered “meta”cognition (Carruthers and Ritchie 2012).

For some time, metacognition was considered a predominantly or even uniquely human trait (Metcalfe and Kober 2005). However, assuming that all extant traits have been shaped by evolution, it seems reasonable to expect to find bases or precursors of metacognition in other species, particularly those that are phylogenetically closest to humans. For this reason, a substantial body of research has addressed the question of metacognition in primates.

Understanding metacognition in nonhuman primates (hereafter, NHPs) is important for several reasons. It can shed light on the evolutionary origins of metacognitive capacities, providing clues about adaptive significance and selective pressures driving their emergence. It can also strengthen inferences about the evolutionary trajectory of metacognition and possible precursors in ancestral lineages, while clarifying continuities in cognitive abilities across species. Investigating metacognition in NHPs can also help elucidate possible cases of convergent evolution versus shared ancestral traits. Moreover, language-free protocols developed to study metacognition in NHPs have been used to explore the ontogeny of metacognition in human infants, providing evidence for implicit metacognitive abilities early in life (Sodian et al. 2012).

Over the past 2 decades, the comparative literature on metacognition has grown substantially, motivating the present review. Here, we examine the current state of research on metacognition in NHPs, covering experimental paradigms and empirical findings in great apes, and catarrhine and platyrrhine monkeys. We also consider proposed alternative explanations of nonhuman metacognitive-like behaviors, discuss field experiments and individual differences, before concluding on future directions. By synthesizing existing knowledge and highlighting unresolved questions, we hope to provide a useful starting point for future research.

## Methods employed to study metacognition in nonhuman animals

Metacognition in humans has typically been studied by directly asking participants to verbally report on their mental states. Since other species lack this ability, researchers have developed paradigms based on nonlinguistic behaviors that could reflect metacognitive processes. To date, most studies have focused on one of the following outputs: avoiding difficult or unsolvable problems, seeking out missing or clearer information, and gambling on success.

Table 1 presents the most commonly used paradigms, with brief descriptions of methods, advantages and possible disadvantages.

**Table 1:** Paradigms used to test nonhuman metacognition

Paradigm	Description	Advantages	Disadvantages
Escape/uncertain response paradigm	Subject presented with a memory or discrimination task of varying difficulty; option to escape difficult trials	Precise control and flexibility, diverse cognitive domain exploration	Intensive training required, potential cue association learning^a^, lack of ecological relevance
Information-seeking paradigm —tubes task	Subject faced with task where crucial information may be missing/ambiguous; option to seek additional information	Naturalistic approach, minimal training, reduced associative learning, detailed analysis of information-seeking behavior	Additional test conditions needed to control for alternative explanations, notably response competition^b^
Pre- and post-trial confidence judgments—escape response/gambling paradigms	Following stimulus presentation, the option to escape or gamble is presented before (pre-trial) or after (post-trial) the memory/discrimination test	Eliminates concerns about response competition and behavioral cues, provides naturalistic assessment, precise control over confidence judgments	Intensive training required, potential cue associations, may lack ecological validity
Post-trial confidence judgments—confidence movements	After completing the test, subject must move to a different location before receiving feedback to obtain reward for successful trial	Naturalistic assessment, eliminates concerns about response competition and behavioral cues, minimal training	May lack precision and control compared to other paradigms

^a^See Alternative explanations section, Associative learning for more explanation of cue association learning

^b^See Alternative explanations section, Response competition for more explanation on response competition

While tests employing uncertainty response paradigms are most often computer-controlled, a few studies have tested apes using a more naturalistic version, in which a piece of food is hidden under one of several cups (Suda-King et al. 2013). The subject can then attempt to find the reward or choose a guaranteed but less desirable reward instead. Information-seeking paradigms also exist in computerized versions, where the subject is given the option to review a sample or ask for hints before answering a memory/discrimination test (Beran and Smith 2011). In this case, the advantages and disadvantages listed in Table 1 would be the same as those for the escape/uncertainty response paradigm.

Studies using the above-mentioned paradigms have contributed to our understanding of metacognitive-like abilities in other species, with researchers choosing the most suitable approach based on their specific research questions and the desired level of ecological validity. As no method is completely immune to non-metacognitive explanations, it would be ideal to test the same species using a variety of protocols to see if performances converge on a metacognitive account. This approach involves employing a range of metacognitive tasks (e.g., escape response, information-seeking, and confidence judgment paradigms), each targeting different non-metacognitive explanations. However, because the protocols used can vary significantly, animals might use different strategies across tasks, which could confound our interpretations. Another strategy to infer metacognition could be to implement task-switching paradigms where animals must apply metacognitive strategies across different tasks within the same experimental session. This could help determine if they are using a flexible, generalizable metacognitive strategy rather than task-specific ones.

In addition, it would be desirable to couple those approaches with a model-based strategy. Developing computational models that simulate metacognitive processes and predict performance across different tasks can provide further insights. By comparing the model’s predictions with actual performance, we can infer the likelihood of metacognitive mechanisms being at work.

### Alternative explanations

As discussed above, none of the paradigms presented in Table 1 is entirely free from non-metacognitive explanations. Below is an overview of the most common alternative mechanisms proposed to explain animals’ metacognitive-like behaviors, along with some methods that can be used to control for them.

### Associative learning

To address early concerns about whether animals were genuinely exhibiting metacognition or merely learning how to respond through association and reinforcement (Crystal and Foote 2009; Le Pelley 2012; Smith et al. 2008; Staddon et al. 2007; Jozefowiez et al. 2009a, b), some authors explored whether the escape response generalizes across different tasks and stimulus sets (Brown et al. 2017; Kornell et al. 2007; Templer and Hampton 2012; Washburn et al. 2006). In some studies, trial-by-trial feedback was replaced by deferred feedback, wherein subjects received all rewards and penalty timeouts for a set of several trials (Smith et al. 2006; Couchman et al. 2010). In others, uncertainty monitoring was evaluated in abstract situations involving metacognitive judgments about memory or learning (Kornell et al. 2007; Morgan et al. 2014; Suda-King 2008; Suda-King et al. 2013; Templer and Hampton 2012; Washburn et al. 2006, 2010). Despite considerable efforts to control for associative learning, leading to a consensus that low-level associative learning processes cannot fully explain the metacognitive-like behavior displayed by NHPs, it should be kept in mind that humans might in fact rely on external cues when making metacognitive judgments, which brings into question the appropriateness of automatically excluding metacognitive processes in nonhuman animals when external cues are involved.

### Response competition

Another proposed alternative mechanism is response competition. When the secondary metacognitive response (escaping the test or seeking additional information) competes with the primary response (solving the memory or discrimination test), animals may default to the metacognitive response when they do not hold the answer to the primary test because of weak motivation to choose (i.e., select a particular tube or match a stimulus). To control for response competition, confidence judgments can be made either before or after a trial. In pre-trial confidence judgment paradigms, subjects are given the option to decline the trial, seek information, or bet on the certainty of their choice *before* the primary test, meaning that they do not yet have access to any trial-specific sensations or cues. Conversely, in post-trial confidence judgment paradigms, subjects have the option to bet on the certainty of their choice *after* completing the test, allowing them to rely on sensations or cognitive assessments experienced during the test to inform their confidence judgment. Although prospective and retrospective metacognitive judgments seem to rely on different mechanisms (Fleming et al. 2016; Goto and Watanabe 2012), both pre- and post-trial confidence judgments paradigms have been used to address concerns about response competition. In both cases, the secondary metacognitive response is no longer competing with the primary test response. Yet, apes (Beran et al. 2015), macaques (Basile et al. 2015; Beran et al. 2015; Ferrigno et al. 2017; Fujita 2009; Hampton 2001; Kornell et al. 2007; Morgan et al. 2014) and even capuchin monkeys (Smith et al. 2020) have demonstrated the ability to accurately express confidence or doubt in this situation.

Response competition can also be controlled by varying the attractiveness of rewards. If subjects’ metacognitive response (i.e., seeking information or declining a trial) is influenced by response competition mechanisms, a higher value reward should strengthen the tendency to immediately answer the test (e.g., select a tube in the tubes task or a stimulus in a match-to-sample task). Consequently, the tendency to seek information or decline should be lower in the high-value reward condition compared to the low-value reward condition. However, when tested on the tubes task with rewards of varying attractiveness, apes and macaques did not show the predicted pattern (Call 2010; Marsh and MacDonald 2012a; Subias et al. 2024a).

### Risk appraisal and curiosity/anxiety

According to Carruthers and Williams (2019), animal behavior in uncertainty monitoring tasks can be explained by first-order appraisals of risk or by affective states such as curiosity or anxiety. They propose that animals tested on uncertainty paradigms may make decisions based on their assessment of the risks involved, without necessarily being aware of their own mental states or uncertainty. Concerning information-seeking, it may be triggered by a feeling of curiosity induced by ignorance. Similarly, in situations of uncertainly, animals might feel anxious (Carruthers and Ritchie 2012; Carruthers 2008). Whether they feel curious or anxious, animals may merely react to their affective state without being aware of their (lack of) knowledge. This comes done to the following issue:

### Being in a state vs. knowing that one is in a state

For Perner (2012), the biggest challenge to attempt to detect metacognition based on behavioral indicators is the difficulty in distinguishing between *being in a state* and *knowing that one is in a state*. “Being in a state” refers to the primary or first-order mental state an individual experiences, such as feeling uncertain or having a preference for a particular choice. By contrast, “knowing that one is in a state” relates to a higher order—or metacognitive— state; i.e., it is a self-reflective or self-aware state. While animals may demonstrate adaptive responses to uncertainty, it is difficult to determine if they are aware of being uncertain.

One way to approach this issue is through neuropsychological and neuroimaging techniques. Several studies (see Catarrhine monkeys section for more detail) have demonstrated a distinction between memory or perception and the metajudgments made by dlPCF-impaired monkeys (Cai et al. 2022; Hampton et al. 2020; Kwok et al. 2019). This suggests that, despite being in a state of knowing, monkeys behaved as if they were not. Such results parallel observations in humans (Lapate et al. 2020) and support the notion of procedural metacognition in rhesus macaques. Similarly, their sensitivity to metacognitive illusion (Ferrigno et al. 2017) indicates that monkeys may be in a state of knowing or not knowing, yet behave otherwise by overestimating or underestimating their performance based on indirect cues (cues that are not the memory itself, for example in a match-to-sample task, the degree of similarity between stimuli).

The mixture of positive and negative results across species has also been used to challenge non-metacognitive explanations (Call 2012). The authors who argue that metacognition is a uniquely human trait counter that differences between species can be explained by distinct cognitive styles or the methods employed to test metacognition. The issue remains an important one for comparative cognitive science to address in future studies.

## Phylogenetic comparison

### Great apes

**Fig. 1:**
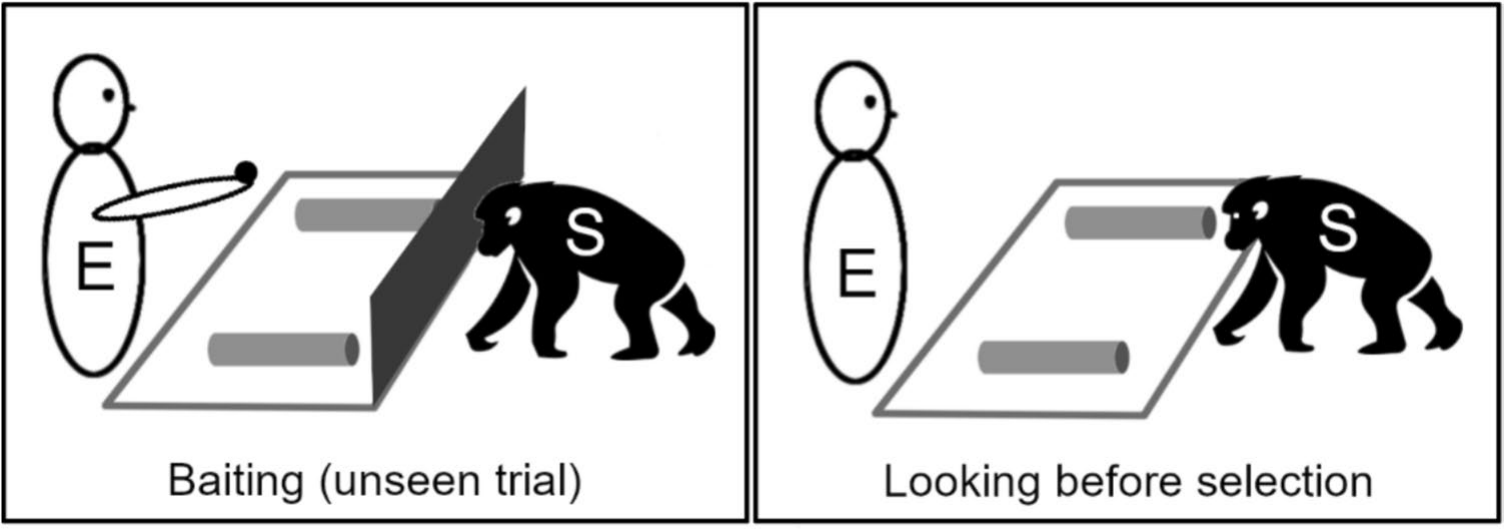
Basic tubes test setup (E experimenter, S subject) as first introduced by Call and Carpenter (2001)

Individuals of all great ape species have been tested for metacognition, with results indicating some degree of monitoring and control over cognitive processes. The first such study is often credited to Premack and Woodruff (1978) in their research with the chimpanzee named Sarah. They explored theory of mind by presenting Sarah with tasks where she had to choose between an immediate reward and a delayed but higher value reward, each in an opaque container. Sarah could peek into one container before choosing, and she tended to do so when uncertain of which container held the higher value reward, suggesting that awareness of her uncertainty guided her choices. Subsequent studies replicated these findings using the tubes task. In this task, the subject must locate a food reward hidden in one of several opaque tubes (or under cups, in some versions). During some trials, the experimenter puts the reward in place in plain sight of the subject, whereas in others, this act is hidden behind an opaque barrier (Fig. 1). Therefore, the subject’s knowledge state can be manipulated by creating situations where they know the reward’s location and others where they do not. Alternatively, combinations of opaque and transparent containers can be used. The crucial aspect of the tubes task is that the subject can bend down to peer through the tubes (or under the cups) and inspect their contents before making a choice; subjects with metacognitive ability are expected to do so when they lack prior knowledge regarding the reward location.

Tested in the tubes task, chimpanzees, bonobos, orangutans, and gorillas have all shown a tendency to look more often inside tubes when they do not know which tube holds the reward compared to when they know (Call and Carpenter 2001; Call 2010; Marsh and MacDonald 2012a, b). When asked to name an item contained in an opaque box, language-trained chimpanzees displayed similar information-seeking behavior, checking the box content before answering when ignorant of the item nature (Beran et al. 2013). These studies also revealed that apes utilize efficient search strategies and that subjects who are able to use auditory information to track rewards inside a container tend to look less when auditory information regarding the location of the reward was given (e.g., when the baited tube was shaken, causing the food to rattle inside it) (Call 2010). This finding contradicts the suggestion that subjects might simply visually search their surroundings until they locate the reward and then choose the correct alternative—the “general search hypothesis” (Hampton et al. 2004; Kornell et al. 2007).

Likewise, the general search hypothesis could be dismissed if subjects were observed solving the tubes task through inference by exclusion. For instance, they might select a third tube without checking its contents after noticing that the first two were empty. However, apes rarely use such inferential reasoning, especially when the number of possible locations increases, which might tax their ability to reason this way (Call and Carpenter 2001; Call 2010; Gazes et al. 2023; Marsh and Macdonald 2012a; Perdue et al. 2018). Alternatively, information obtained through inference might not be accessible to monitoring. In other words, it might be too demanding for apes to combine inferential reasoning and metacognition.

Together, the studies described above have revealed that great apes’ decisions about seeking information depend on multiple factors, including the effort required, reward qualities, and the relative risks associated with errors (Call 2010; Gazes et al. 2023; Mulcahy 2016; Perdue et al. 2018). In addition, the apes demonstrate information-seeking in tool-use contexts, tool locations and with regard to tool properties (Bohn et al. 2017; Mulcahy 2016; Perdue et al. 2018). The flexibility of great apes’ seeking behavior dismisses several non-metacognitive explanations. For instance, if apes were simply searching their environment until they spotted food or using cue associations to determine when to look, increasing the effort required for seeking (or the risk of making an error) should have no effect on their seeking behavior, or a similar effect regardless of whether they had witnessed the baiting. Instead, when the effort increases, apes significantly reduce looking when it is unnecessary but maintain a high rate of looking when it is necessary (Call 2010; Gazes et al. 2023). Response competition remains a valid explanation in most tubes task studies. To our knowledge, only one published study with great apes has attempted to test this hypothesis using rewards of varying attractiveness, as mentioned in “Alternative explanations” section (Call 2010). When a more attractive reward was at stake, apes increased unnecessary looking instead of reducing it, which contradicts the predictions of the response competition hypothesis. However, we should remain cautious, as apes may still be solving the task through response competition mechanisms and may look from time to time when they should not (and more when a more attractive reward is at stake) because looking at the reward may be intrinsically pleasant.

**Fig. 2:**
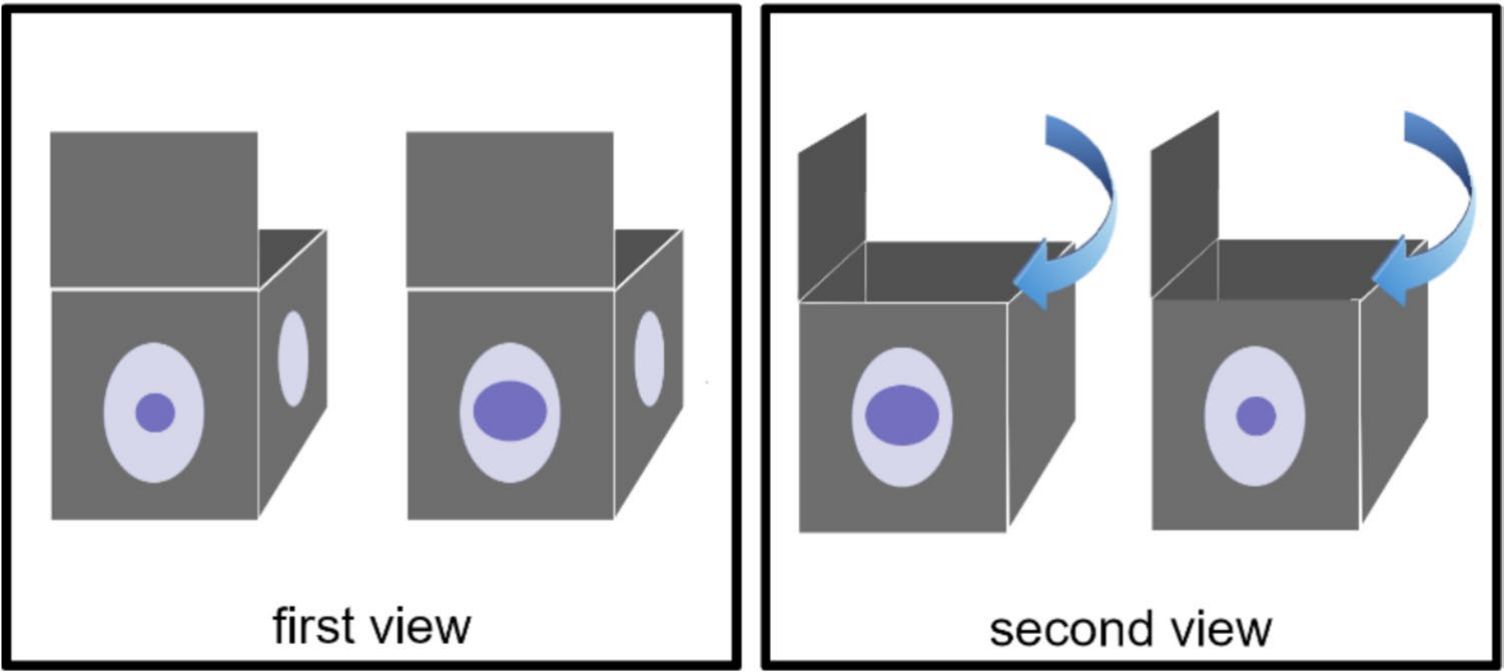
Schematic of the apparatus used by O’Madagain et al. (2022) to test belief revision. On the “first view”, one reward looks bigger than the other. On the “second view”, the boxes have been rotated and the opposite reward appears bigger (in the “conflicting” condition)

O’Madagain et al. (2022) recently took a different approach by comparing chimpanzees’ ability to question beliefs with that of 5- and 3-year-old human children. Participants were presented with two boxes, each equipped with windows on the sides. Both boxes contained a reward, with one being larger (and therefore more preferred) than the other. Initially, participants made a choice between the two boxes. Subsequently, the boxes were rotated 90 degrees to reveal a different view of their contents, where the rewards could appear larger or smaller due to the use of magnifying/minimizing lenses (Fig. 2). When the appearance of the rewards in the second view conflicted with their initial choice, 5-year-olds (but not 3-year-olds) and apes both sought additional information by peeking inside the boxes from the top before making their final choice. In a second experiment, the content of the boxes was disclosed to a partner (chimpanzee or human, depending on the species tested), who then pointed to one (aiming to identify the largest reward). When the social partner’s choice conflicted with the initial choice of the participant, children of all ages tended to reassess their beliefs by peeking inside the boxes before making their final choice, while chimpanzees disregarded the disagreement. The authors argue that although great apes may be able to rationally monitor their decisions, the capacity to solve problems socially might set humans apart from other species.

Some studies have explored apes’ metacognitive-like abilities using the escape response paradigm. Suda-King (2008) and Suda-King et al. (2013) tested orangutans and gorillas, respectively, on a spatial memory task where they had to remember the location of a preferred food reward (two grapes) hidden under one of several cups. Alternatively, apes could choose to escape the test by selecting a “safe” cup that always contained a smaller amount of food (one grape). Orangutans and gorillas tended to escape trials (i.e., select the one grape cup) when valuable information was missing. However, in each experiment, only one subject avoided trials in which the escape option was presented prior to the memory test, suggesting that the success of the others might be explained by non-metacognitive mechanisms such as response competition or associative learning. In contrast, great apes may be more successful on post-trial confidence judgment paradigm. In an experiment by Beran et al. (2015), chimpanzees performed a computerized matching-to-sample task, requiring them to leave the computer apparatus before receiving any feedback and walk to the location where food would be dispensed if their response was correct. If they were not at that location on time, the reward was lost. James et al. (2021) used a similar protocol to test 3- to 5-year-old children. In both studies, chimpanzees and children left the test area and walked to the dispensing area more often on trials that were correctly completed than on those that were not, suggesting the ability to assess their memory and correctness in each trial.

In humans, behaviors linked to uncertainty, such as longer response times, hesitation, or hand-wavering, contribute to metacognitive judgment (Wokke et al. 2020). Recently, Allritz et al. (2021) studied three chimpanzees during experiments on social learning and transitive inference using a touchscreen task. As task difficulty increased, the chimpanzees exhibited more hand-wavering between the stimuli on the screen, similar to humans, suggesting a shared experience of and response to uncertainty. Wavering is an external public cue, a type of cue that is observable by anyone, not only the subject. This contrast with private cues, which are internal and only observable by the subject themselves (for example, a memory). According to Hampton (2009), public cues do not qualify as metacognitive, whereas private cues entail metacognition. While hand-wavering is a public cue, Allritz et al. (2021) argue that if individuals respond to their self-generated behavioral cues with adaptive second-order behaviors, metacognition may be inferred.

It is generally accepted that demonstration of metacognition should meet four criteria (Hampton 2009):

1. There must be a primary, observable behavior that can be scored for its accuracy (for example, solving a problem or performing a memory test could be a primary observable behavior. Accuracy or efficiency could be scored by assessing the time taken to solve the task or the percentage of correctly answered trials).

2. There must be variations in performance, and thus, variations in the accuracy or efficiency of the primary behavior (how well the animal is performing on a given task).

3. The animal must elicit a secondary behavior (for example, skipping or studying longer on difficult trials), the goal of which is to regulate the primary behavior.

4. The secondary behavior must benefit performance in the primary behavior (skipping trials or studying longer should benefit the animal’s performance on the task).

While Allritz et al.’s (2021) study did not feature an option for the animal to elicit a secondary behavior, their argument is that if subjects are able to use their hand-wavering as a cue to, for example, skip trials that they would overwise fail, then metacognition may be involved. Future studies could usefully explore in more detail the relationship between subtle behavioral cues of uncertainty and information-seeking or escape responses, with wavering serving as an indirect, untrained, non-invasive proxy for uncertainty or lack of confidence.

No significant species differences among the great apes have been found in these tasks, though gorillas sometimes exhibit slightly divergent response patterns, possibly due to greater sensitivity to physical effort or higher confidence levels (Call 2010; Gazes et al. 2023).

### Catarrhine monkeys

Most laboratory studies on metacognition in monkeys have focused on rhesus macaques (*Macaca mulatta*). Across various paradigms, rhesus have demonstrated strong evidence of an ability to accurately monitor some memories. Initial experiments utilizing the “escape response” paradigm revealed that monkeys selectively avoided tests when their memory was poor but engaged and performed accurately when their memory was good (Hampton 2001). Subsequent tests confirmed these findings in generalization tests: in delayed match-to-sample tasks, monkeys tended to decline trials after longer intervals or when not provided with a sample to remember (Brown et al. 2017; Smith et al. 2010; Templer and Hampton 2012; Washburn et al. 2006).

**Fig. 3:**
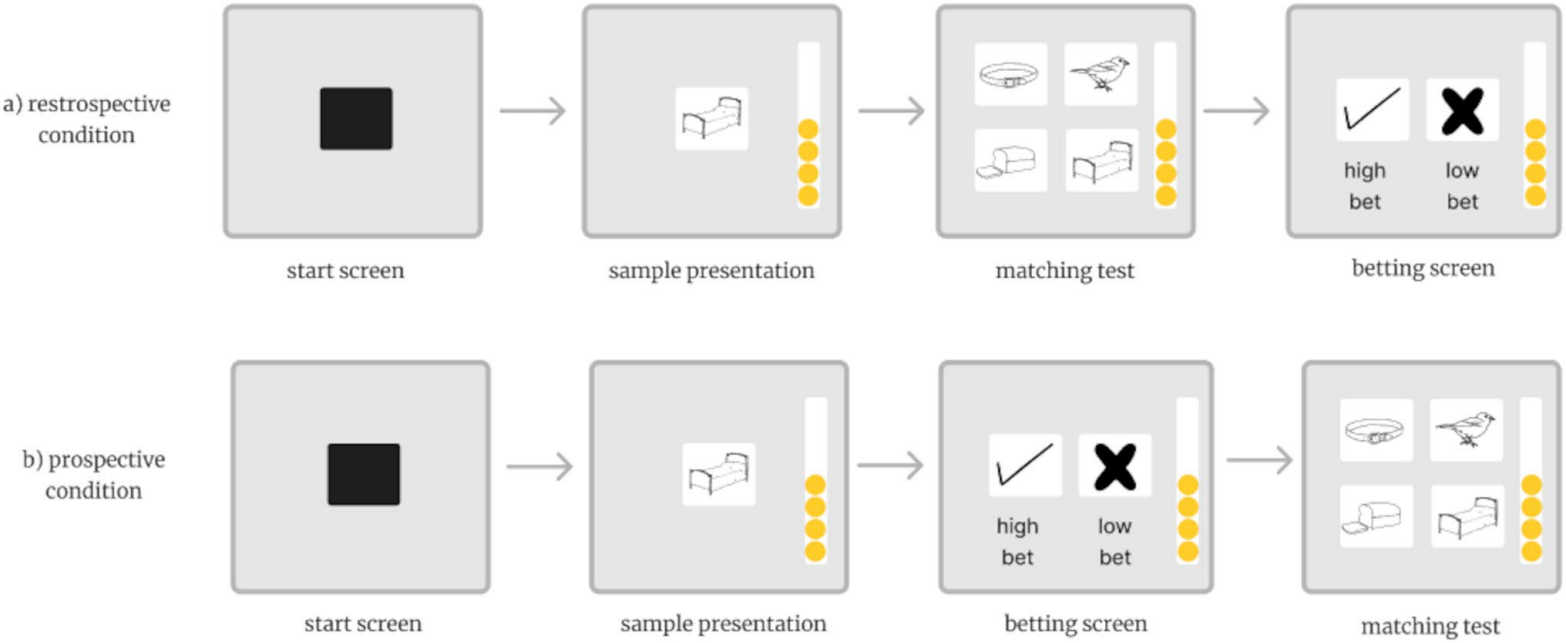
Simplified schematic of protocol using a betting paradigm to test retrospective (**a**) and prospective (**b**) judgment in monkeys. The white band with yellow circles on the right edge represents a token bank that can be filled and emptied depending on the subject’s accuracy on the test and betting choice. When the bank is full, the subject earns a food reward

If in most studies the metacognitive judgment is concurrent with the primary task, meaning that the option to escape a test or seek information is presented at the same time as the memory or discrimination test, uncertainty tests can also be given before (pre-trial) or after (post-trial) the primary test. Metacognitive judgment can thus be prospective and retrospective (Terrace and Son 2009). In one such study, rhesus monkeys were tasked with “betting” on the accuracy of their response (Table 1, Gambling paradigm) in a delayed matching-to-sample test (Morgan et al. 2014). They were presented with two icons representing “low” or “high” risk. Choosing the low-risk icon guaranteed a small reward regardless of their performance, while selecting the high-risk icon offered a larger gain for successful completion of the trial but incurred a substantial loss for an incorrect response (Fig. 3). Monkeys generally took risks and opted to bet high on successful trials, while choosing the small, guaranteed reward more often when they failed, illustrating retrospective as well as prospective judgments of confidence.

Even when memory was disrupted due to transcranial magnetic stimulation (TMS), a monkey still displayed proper use of the escape response, thereby ruling out external cues as an explanation for its behavior (Washburn et al. 2010). In addition, besides accurately assessing the presence of a memory, monkeys also accurately gauged their ability to recall the order of a sequence of events (i.e., report which of two images from a list had appeared first during study; Templer et al. 2018).

As well as avoiding trials when uncertain, rhesus monkeys also exhibit appropriate information-seeking behavior when tested on computerized delayed matching-to-sample tasks (Beran and Smith 2011) or the tubes task (Hampton et al. 2004). Six possible explanations of the results of those studies, beyond memory monitoring, were explored and ruled out, supporting the argument that monkeys utilize memory strength as a discriminative cue for memory monitoring (Basile et al. 2015). Some monkeys have even shown spontaneous memory monitoring without training; for example, Hampton and Hampstead (2006) observed a monkey displaying signs of frustration before receiving feedback on failed memory tests.

Further evidence that monkeys can engage in some form of metacognitive process is their susceptibility to false judgments—also referred to as metacognitive illusions—similar to humans (Ferrigno et al. 2019; 2017). A metacognitive illusion occurs when someone’s understanding or perception of their own thinking abilities or knowledge is incorrect or biased, leading to a false sense of confidence or uncertainty. In a match-to-sample task where rhesus monkeys were presented with high- or low-contrast stimuli, they exhibited different levels of confidence in their responses based on the contrast of the stimuli. However, the stimuli contrast did not affect their accuracy (Ferrigno et al. 2017). Therefore, the monkeys displayed what might be a dissociation between memory accuracy and confidence judgments. Similarly, neurobiological studies have revealed distinct memory and confidence judgment functions in monkeys. Temporary deactivation of specific regions within the frontal cortex resulted in impaired retrospective memory assessments, although performance in the actual memory tests, which served as the basis for their confidence judgments, remained unaffected (Miyamoto et al. 2017, 2018).

Studies of monkeys’ ability not only to monitor and control memory but also their perceptions have shown that they tend to choose simpler visual discriminations over more challenging ones (Beran et al. 2006; Brown et al. 2017); furthermore, they can transfer their use of the escape response to new tasks (Kornell et al. 2007). It is noteworthy that competing cognitive load and disruptions to the dorsal part of the lateral prefrontal cortex (dlPFC) impair confidence judgment while leaving monkeys’ accuracy on the primary perceptual task intact (Cai et al. 2022; Kwok et al. 2019; Middlebrooks and Sommer 2012; Smith et al. 2013), suggesting that the dlPFC is involved in their confidence judgments about perceptual experiences and memory. Interestingly, in Cai et al. (2022), the monkeys did not use reaction time as a cue to make their judgment unless dlPFC activity was disrupted. This suggests a shift in the monkeys’ strategy, relying on external cues only when introspection is not possible. Moreover, comparison of how similar the monkeys judged their performance within a task (testing either memory or perception) and across tasks revealed greater within- than across-judgment consistency, supporting the idea that possible metacognitive abilities are influenced by the specific task being performed. Metacognition in monkeys might be domain-specific, as suspected in humans (Morales et al. 2018). These neurobiological studies suggest that monkeys engage in monitoring and control processes related to perception or memory. According to Hampton et al. (2020), if these processes can be monitored, they appear to be explicit.

Most metacognition experiments have treated “information” as an all-or-none concept, but a few studies have attempted to explore how monitoring subtly evolving cognitive states influences the desire for information. When the quantity of accessible information in a classification task varied (i.e., monkeys classified images as birds, fish, flowers, or people, with the to-be-classified images not visible at the beginning of a trial), rhesus monkeys adjusted their information-seeking efforts accordingly, making more or fewer “revelation” responses depending on the difference between information acquired and information needed (Brady and Hampton 2021; Tu et al. 2015). When testing rhesus monkeys on a four-choice, match-to-sample memory task with the option to decline the trial and review the sample, Brown et al. (2019) found that when metacognitive judgment was available before the test, monkeys were less accurate compared to when the judgment was concurrent with the test. This shows that both working memory and stimulus-evoked familiarity influence confidence judgments, and that—similarly to humans—prospective and retrospective judgment might be dissociated in monkeys, involving different neural systems (Fleming and Dolan 2012; Siedlecka et al. 2016).

Other cercopithecine species tested for metacognitive abilities include lion-tailed macaques (*Macaca silenus*, Marsh 2014), Japanese macaques (*Macaca fuscata*, Subias et al. 2024a, b), and Guinea baboons (*Papio papio*, Malassis et al. 2015). In all three studies, monkeys appropriately sought information when ignorant, but macaques also unnecessarily inspected the containers when the reward location could be logically inferred. So far, macaques have shown no clear evidence of adjusting their information-seeking behavior based on inference by exclusion. However, the limited number of studies prevents any definitive conclusions. As noted earlier, similar studies with apes indicate that they sometimes used exclusion inference, though their performance declined as the number of possible locations increased. Notably, tubes task studies with macaques involved more locations—four (Hampton et al. 2004; Subias et al. 2024a, b) or three (Marsh 2014). Similarly, with four tubes, gorillas also failed to use exclusion to terminate their search early (Gazes et al. 2023). Whether macaques’ failure to use exclusion inference is due to the increased cognitive demands of the tasks they have been subjected to or reflects a true limitation in their abilities remains unknown. Interestingly, like great apes, Japanese macaques showed some sensitivity to the cost of seeking information and the quality of the reward at stake. While additional studies are required for confirmation, the evidence to date suggest that members of different branches of the Cercopithecidae share similar abilities.

Research has provided multiple lines of converging evidence for the monitoring and control of some cognitive processes in catarrhine monkeys, with most studies having been done on rhesus macaques. Unlike great apes, in whom individual differences in performance are often marked (see Individual differences section), most macaques tested so far appear to exhibit behavioral patterns consistent with procedural metacognition. This divergence might be explained by methodological differences: studies testing apes usually involve less training and fewer test trials. In addition, apes have been mainly tested on information-seeking paradigms using some version of the tubes task, whereas macaques have been tested on escape response paradigms, often involving extensive computerized training.

### Platyrrhine monkeys

Investigations of metacognition among Platyrrhini have focused mainly on tufted capuchin monkeys (*Sapajus apella*), with inconsistent results. For instance, Fujita (2009) found that two capuchins tested on a delayed matching-to-sample task more frequently escaped trials with longer delay and decreased accuracy; however, they still accepted trials with a high chance of error, and one showed higher accuracy on chosen than on forced trials. Similarly, when given the choice, capuchins selected the task at which they performed better, but their accuracy was not significantly higher than in trials where no choice was given (Takagi and Fujita 2018).

Capuchins’ use of an uncertainty response (UR) was evaluated in a fine visual discrimination task (Beran et al. 2009). Monkeys were trained on two tasks: one involved pixel discrimination with sparse, intermediate, and dense conditions, and another with sparse and dense conditions and the option of an “uncertain” response (UR). Monkeys were rewarded only for categorizing pixel boxes correctly, not for the UR. Although they used the “intermediate” response effectively, they rarely used the UR, even when faced with penalties for incorrect responses. Subsequent studies confirmed that capuchins appear reluctant to employ the UR, unlike macaques (Beran et al. 2014; Perdue et al. 2015). These divergent performances indicate that the behaviors suggestive of metacognition in macaques using the UR is unlikely to be due to them treating it as another categorization response, as capuchins did not employ it in this way. However, Beran et al. (2016) found that changes in reward contingencies led to capuchins showing increased UR usage in trials with lower success probabilities, albeit at a relatively low rate.

Studies employing information-seeking paradigms have found limited evidence for metacognition in capuchins. Paukner et al. (2006) observed irrational information-seeking behavior, with capuchins needlessly looking into transparent tubes and also opaque, bent tubes that could never yield the necessary information. In Marsh and MacDonald’s (2012b) study, during the opaque versus transparent task, one orangutan exhibited a pattern of unnecessary looking when the reward was placed under the transparent cup. However, as a group, great apes looked significantly less often when the food was placed under the transparent cup, contrasting with the results for capuchins in Paukner et al. (2006). In a later study (Vining and Marsh 2015), capuchins performed better when the number of cups was reduced, suggesting that their failures in information-seeking tasks may be partially due to an excessively high cognitive load caused by an increased number of possible locations, and in Basile et al.’s (2008) study, three out of five capuchins looked significantly less often in visible compared to hidden trials. Although capuchins appear more inclined to investigate tubes or cups when lacking information regarding their contents, they still tend to inspect them unnecessarily (Basile et al. 2008; Vining and Marsh 2015). And when the effort required to look inside the containers was increased by lowering the tray on which the device rested, capuchin monkeys showed less looking regardless of whether they observed the baiting, in contrast to great apes and macaques who only reduced unnecessary looking (Call 2010; Gazes et al. 2023; Marsh and MacDonald 2012b; Subias et al. 2024a). Vining and Marsh (2015) suggested that capuchin monkeys may possess a rudimentary metacognitive capacity when dealing with “externally derived sensory information,” such as food being left in a location. However, their ability to handle cognitive information, particularly abstract uncertainty arising from not knowing a discrimination response, appears limited. Computerized testing revealed that capuchin monkeys engaged in information-seeking when appropriate, just like macaques (Beran and Smith 2011), but whereas macaques displayed flexible and varied forms of information management, capuchins did not.

The only study to date that has tested capuchins for post-trial confidence judgment using movement (Smith et al. 2020) produced positive results. The experimental setup involved a computerized memory test in one location, with rewards for correct responses dispensed in a separate room, similarly to a situation used with apes (Beran et al. 2015). Analyses of the monkeys’ response times and movements between these locations before receiving feedback indicated an ability to monitor confidence in their responses, although their confidence movements were less precise and flexible than those of chimpanzees.

In summary, capuchins demonstrate basic metacognitive-like abilities, but not to the same degree of complexity and exactitude as great apes and macaques. They show limited transfer of the uncertain response to other tests, and though they respond metacognitively with external, salient stimuli, they struggle with more abstract stimuli. Capuchins might lack the ability to monitor and control cognitive processes as effectively as great apes and macaques, or the methods used to test them may not have been sensitive enough to fully capture their potential metacognitive abilities (Smith et al. 2018). Support for the latter view comes from capuchins’ relatively strong risk-tolerance, as highlighted in some of the studies cited above (Beran et al. 2014, 2016). Attention, impulsiveness, motivation, and perception are among the factors that may interact with the experimental designs used to study metacognition.

We know of only one study that has attempted to assess metacognitive abilities in lemurs. Taylor et al. (2020) presented lemurs (red-bellied: *Eulemur rubriventer*, ring-tailed: *Lemur catta*, black and white ruffed: *Varecia variegata*) using a tubes task, with no evidence of metacognitive responses. However, more research is required before more definitive conclusions can be drawn regarding metacognition in strepsirrhine primates, with due attention to methodological issues such as attentiveness to the baiting, failure to understand transparency (Taylor et al. employed transparent tubes to create trials in which looking was unnecessary), and the “cost” of looking being too low (i.e., looking inside the tube was too easy).

Table 2 summarizes all NHP species tested for metacognition to date, along with the methods used and the outcomes, based on the strength of the evidence.

**Table 2:** Methods employed to study metacognition in nonhuman primate species

Species		Methods	Degree of success on metacognition testing*
Great apes	Chimpanzees	Information-seeking—tubes task	High
		Post-trial confidence judgment—confidence movements	
		Other: behavioral observations	
	Bonobos	Information-seeking—tubes task	Moderate
	Orangutans	Escape/uncertain response—spatial memory task using cups	High
		Information-seeking—tubes task	
	Gorillas	Escape/uncertain response—spatial memory task using cups	High
		Information-seeking—tubes task	
Catarrhine monkeys	Rhesus macaques	Escape/uncertain response—computerized task	High
		Information-seeking—tubes and computerized tasks	
		Pre- and post-trial confidence judgments—escape response/gambling	
		Other: behavioral observations	
	Lion-tailed macaques	Information-seeking—tubes task	Moderate
	Japanese macaques	Information-seeking—tubes task	Moderate
	Guinea baboons	Information-seeking—computerized task	Moderate
Platyrrhine monkeys	Tufted capuchins	Escape/uncertain response—computerized task	Moderate
		Information-seeking—tubes and computerized tasks	
		Post-trial confidence judgment—confidence movements	
Strepsirrhines	Red-bellied lemurs Ring-tailed lemurs Black and white ruffed lemurs	Information-seeking—tubes task	Low

*“High” refers to success on multiple paradigms with appropriate control conditions. “Moderate” refers to mixed positive and negative results, or tests using only one paradigm. “Low” refers to few or no positive results

The performance differences observed among lemurs, capuchins, rhesus macaques, and great apes have led some researchers to suggest that metacognition may not be a universal ability among primates, or it may not be equally developed among them (Hampton 2019; Taylor et al. 2020; Smith et al. 2018). However, strong conclusions in this regard appear premature, given the current lack of understanding about the origins of these differences. Future research employing standardized paradigms—that is, experimental methods and procedures that are consistent and uniform across different studies—would facilitate more meaningful comparisons. However, it is also desirable to design experiments that are better aligned with species-specific traits. For instance, testing lemurs with olfactory or auditory instead of visual stimuli might be advantageous, considering the importance of scents and sounds in their everyday lives.

### Field experiments

Most investigations of metacognition in nonhumans have been done in laboratory settings, with only two published studies so far attempted in the wild. These have yielded results similar to those obtained under controlled laboratory conditions. Rosati and Santos (2016) and Subias et al. (2024a) assessed information-seeking behavior in wild rhesus macaques and Japanese macaques, respectively. In the first study, groups of rhesus macaques were presented with two opaque tubes put on the ground and arranged in a V shape pointing toward the subject with a gap at the V point separating them. After attracting the attention of a nearby monkey, the experimenter placed a piece of food inside one of the tubes at its distal end opening, either visibly or surreptitiously. Without any training, individual monkeys were given only a single trial (visible or hidden condition) in which they could either directly approach one tube to get the food, or first seek information by approaching the center of the V formation to look inside both tubes. Monkeys in the hidden condition tended to approach the center to look before choosing a tube. In contrast, monkeys in visible condition tended to directly approach the distal end of the baited tube. This study demonstrates that rhesus macaques can show information-seeking behavior without any training. However, this experiment alone does not provide strong evidence of metacognition, as the design lacks sufficient control for alternative explanations such as response competition.

**Fig. 4:**
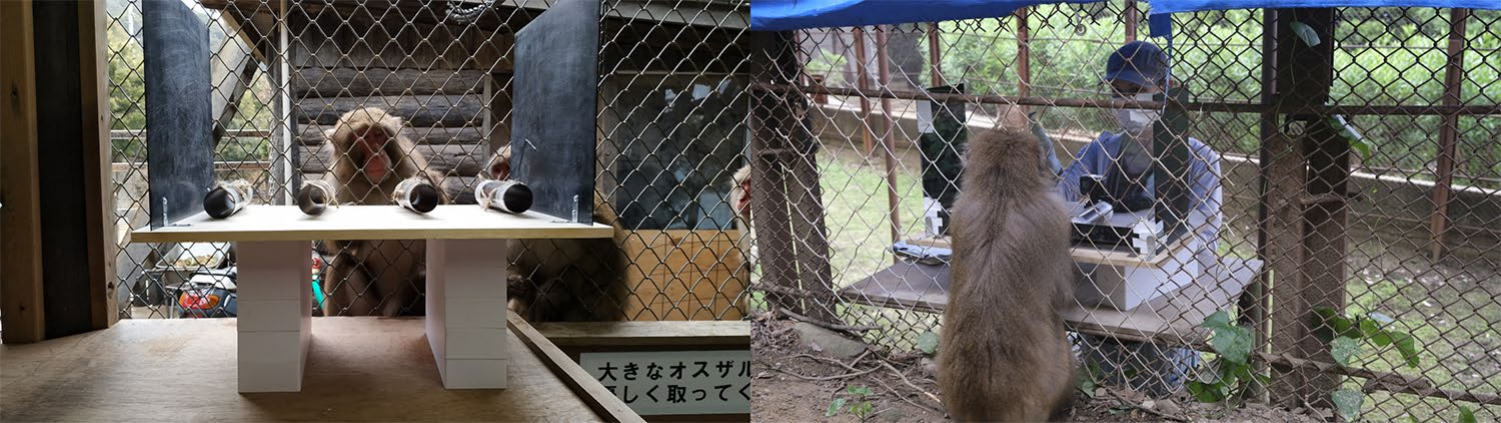
Free-ranging Japanese macaques tested on the tubes task at the Awajishima monkey center (Subias et al. 2024a). Two testing locations were set up, with the experimenter standing inside wire-mesh huts. The white boxes were used to adjust the apparatus height and to manipulate the effort required to look

A tubes test study on free-ranging Japanese macaques enabled a deeper analysis of the cognitive mechanisms behind monkeys’ seeking behavior, by repeated testing of the same subjects and additional conditions (Subias et al. 2024b) (Fig. 4). The findings revealed that the monkeys were significantly more likely to look into tubes when faced with ambiguity regarding the reward’s location. Moreover, most monkeys adjusted their seeking behavior by reducing the number of looks when the effort required to look increased, but only if they had prior knowledge of the reward’s location. These results challenge the notion that monkeys merely form associations between external cues (such as the baiting process) and predetermined responses. In addition, the fact that monkeys adapt their seeking behavior based not only to whether they have seen food but also to the level of effort required for seeking demonstrates that they are not using a simple strategy of “search until food is spotted,” as proposed by the general food search hypothesis. When response competition was evaluated by presenting monkeys with more or less attractive rewards (peanuts or carrots), none showed the expected pattern of reduced looking rate with a more attractive reward. Therefore, neither associative learning, generalized food search strategy, nor response competition mechanisms seem able to fully explain the differential information-seeking of Japanese macaques. Together, these studies confirm that behaviors suggestive of metacognitive abilities are not limited to captive macaques with a history of training on cognitive tasks.

Field testing presents logistical challenges and often employs less strict protocols than laboratory experiments. However, it can offer several scientific advantages, including larger sample sizes and the opportunity for long-term, intra- and inter-population comparisons that could shape hypotheses regarding possible functions of metacognition, including questions related to survival and reproductive fitness. In humans, metacognitive performances is known to vary with age, notably declining in older adults (Overhoff et al. 2021), who are more susceptible to negative beliefs about their memory (Gautier et al. 2022). Furthermore, cultural factors may be important, as seen in the more efficient metacognitive evaluation of perceptual decision-making tasks in Chinese than British participants (van der Plas et al. 2022). These findings collectively suggest that metacognitive skills are not uniform and can be influenced by a range of factors. A combination of field and laboratory experiments may be the best approach for reaching a comprehensive understanding of animals’ cognitive capacities from both proximate and ultimate perspectives.

To this end, the next step could involve developing a more efficient experimental apparatus and protocol for data collection in natural settings. An apparatus that can be placed in the environment and left there for the animals to approach and manipulate themselves to obtain food without the intervention of an experimenter would be useful. Hence, several such apparatuses could be placed to allow for the testing of multiple individuals at once.

For example, an apparatus with multiple compartments: choosing to open one compartment will automatically seal the others, but there would be an option to check the content of the compartment (i.e., to engage in metacognition by seeking information). By varying several parameters in addition to the subjects’ knowledge state, such as the value and number of the rewards, the effort required for checking, and the risk of making an error, we may better understand the cognitive mechanisms involved.

It should be acknowledged that placing such apparatuses in an open field where subjects move freely may introduce the possibility of nearby individuals learning by observation. Although this could be viewed as a potential confound, we believe it presents a valuable opportunity to explore the role of social learning in information-seeking behavior. By recording the setup and tracking nearby individuals, we could examine how subjects perform after observing another individual interact with the apparatus. In addition, it would be insightful to assess whether subjects behave differently when being observed compared to when they are alone.

Such an approach may allow testing a larger sample size, facilitating the exploration of how factors such as social learning, social rank, sex, and age influence information-seeking. If a correlation between metacognition and sociality exists, it could lead to the differentiation of potential metacognitive abilities among primates based on their social rank or sex. For instance, it is conceivable that individuals occupying lower social ranks, who are frequently required to suppress their behaviors and employ more intricate strategies to obtain food or reproductive opportunities, may demonstrate a heightened level of metacognition. Field experiments have an important role to play, particularly for addressing issues such as the relationship between metacognition and reproductive fitness.

### Individual differences

It is essential to acknowledge the individual differences often observed in metacognition testing. These differences seem particularly common in cognitive tasks involving great apes. Among the studies discussed earlier, not all subjects exhibited behaviors indicating control consistent with their knowledge state. In a study by Perdue et al. (2018), two of three chimpanzees failed to generalize appropriate tool use across conditions or to infer food locations. In a study by Call and Carpenter (2001), three of six chimpanzees employed an excessive looking strategy, and one of the three orangutans also searched when unnecessary. It is possible that only certain individuals within a species may possess the capability (or willingness) to use metacognition, especially if the perceived benefit of doing so is not substantial.

Focusing on these individual differences can provide valuable insights into the cognitive abilities that constitute metacognition. If specific individuals consistently demonstrate behaviors aligned with metacognitive processes, this variability might support the idea that metacognition is present within the species but manifests selectively based on factors such as cognitive capacity, experience, or motivation. Smith (2005) observed that humans and other primates tested in the escape response paradigm exhibited similar ranges of individual differences, with some individuals never opting for the “escape” option. These differences may arise from variations in cognitive capacities or from differences in personality and preferred strategies. By analyzing individual differences in detail, researchers can gain a more nuanced understanding of how metacognition manifests across different individuals, thus providing further evidence for the existence of metacognition from multiple perspectives. Future studies should make an effort to recognize and analyze these individual differences in detail, and be more cautious when assuming that a species possess metacognition when several subjects failed to exhibit the predicted patterns (Templer 2022; Tomasello 2023). Ultimately, uncovering the cues and mechanisms underlying the various behaviors observed during experimental testing could lead to genuine progress in our understanding of animal cognition.

## Conclusion and prospects

When faced with memory tests or discrimination tasks, great apes and macaques often opt to avoid challenging trials, seek information before responding when uncertain, and accurately gauge their own performances. Carefully controlled experiments have made it possible to counter alternative explanations that are based on associative learning, stimulus aversion, experiment tracking, and response competition, leading to a consensus that mechanisms enabling the monitoring and control of cognitive processes are indeed present, at least in some species (Beran 2019; Call 2012; Couchman et al. 2012; Hampton et al. 2020). Whether these processes qualify as metacognition remains the subject of debate (Carruther and Williams 2019; Comstock 2019; Hampton et al. 2020; Proust 2019).

Despite efforts to refine methods and reject alternative hypotheses, a critical gap in the field of comparative metacognition research is the small number of species tested. Given the apparent existence of intriguing species differences, direct empirical comparisons using standardized methods are desirable to clarify phylogenetic distribution of metacognition.

Schwartz et al. (2023) propose that metacognition may be shaped by a “call for flexibility,” with species inhabiting habitats characterized by fluctuating food sources and social structures being more likely to have evolved metacognitive capacities. Studies of species that are genetically similar but that differ in their natural ecology would be useful for testing this hypothesis. Another avenue we would like to suggest involves the possible role of social tolerance in the development of metacognition. Some studies have suggested that social tolerance may be linked to improved inhibition (Joly et al. 2017; Loyant et al. 2022), a cognitive function closely associated with metacognition (Lysaker et al. 2008; Shimamura 2000). Given this observation, it is plausible that more socially tolerant species, such as Tonkean macaques and bonobos, might exhibit superior metacognitive abilities compared to less tolerant species like rhesus macaques and chimpanzees. Food competition has also been proposed as a driving factor in the evolution of metacognition. Tomasello (2023) suggested that intense food competition among early great apes might have spurred the development of metacognitive and social cognitive skills, including theory of mind (the ability to understand that others’ beliefs, desires, intentions, emotions, and thoughts may differ from one’s own), to better predict others’ behavior in competitive contexts. This hypothesis implies an intimate relationship between social cognition and metacognition, potentially linked to social learning and the intensity of food competition. Comparing the metacognitive performances of highly despotic species with intense food competition to those of more socially tolerant species could help discern whether social tolerance or food competition is more closely associated with metacognition.

Another promising avenue is a greater rapprochement between neurophysiological and behavioral metacognition paradigms. Comstock (2019) advocates a deeper exploration of the neurobiological and genetic underpinnings of metacognition to bolster the case for its existence in animals, but such studies remain scarce due to the numerous challenges they pose.

A less invasive yet potentially informative area of study pertains to metacognitive errors or illusions, which although well studied in humans (Fiechter and Kornell 2021) remain largely neglected in other species. The fallibility of metacognitive judgment may be a human universal, but only one study (Ferrigno et al. 2017) has attempted to investigate metacognitive illusions in NHPs (rhesus monkeys), showing an effect of stimuli fluency that led to a discrepancy between monkeys’ judgment of confidence and their accuracy. Great apes have demonstrated more varied results than rhesus macaques on metacognitive tests, with some individuals not consistently exhibiting metacognitive behavior. This divergence could potentially be attributed to the susceptibility of apes, akin to humans, to fall prey to metacognitive illusions. Similarly, capuchins’ poor performance on metacognition tests has been attributed to a tendency to rely on stimuli fluency (Smith et al. 2018). Those hypotheses needs to be investigated. It would be extremely interesting to test great apes (including humans), Catarrhine, and Platyrrhine monkeys in an experiment similar to that of Ferrigno et al. (2017), evaluating their ability to make accurate judgments in a memory test, and assessing their sensitivity to metacognitive illusions by manipulating the perceptual fluency of stimuli. As have argued several researchers (Beran 2019; Ferrigno et al. 2019; Kornell 2014; Smith et al. 2018), we believe that metacognitive errors provide compelling evidence of animal and human metacognition and offer an opportunity to identify the factors influencing metacognitive judgment.

While the gambling paradigm used by Ferrigno et al. (2019) is impressive and informative, it requires extensive training and may not be applicable to many species. A simpler way to explore metacognitive errors could lie in unnecessary search behavior observed in information-seeking paradigms. In the tubes task studies discussed earlier, while apes and macaques look significantly more often inside the tubes when they have not witnessed the baiting, they still look occasionally when they have witnessed the baiting. Since subjects’ first look is almost always directed toward the baited tube (Call 2010; Subias et al. 2024a), we can assume that they remember the reward’s location, so those unnecessary looks are not due to poor memory. This could constitute a metacognitive error, wherein the animal falsely assumes they do not remember when in fact they do. Call (2010) also proposed that the subject might want to check that they remember correctly, in other words, be cautious, especially when the stakes are high and the cost of checking is low (“passport effect”). Alternatively, looking at food may be intrinsically rewarding (Perner 2012), and the drive to look at the food inside the tube might sometimes override the drive to select the tube.

We explored this hypothesis by testing whether Japanese macaques would look inside a single tube they knew contained food they could not immediately reach (Subias et al. 2024b), and compared their looking behavior to the unnecessary looks they made during a previous tubes task experiment conducted a year earlier. We found that macaques would still look inside the tube even though there was only one tube (and thus, little to no uncertainty regarding the reward’s location), and they looked more when a more attractive reward was at stake. However, monkeys still looked more often in a four-tube situation compared to one tube, and those displaying the highest rate of unnecessary looking during the tubes task (with four tubes) displayed the lowest rate of looking in the one-tube situation, revealing a strong negative correlation. Hence, it seems that “a desire to look at the reward” cannot completely account for macaques’ unnecessary looking behavior in the tubes task.

Nevertheless, it would be premature to disregard this explanation. To reach stronger conclusions, we need to explore unnecessary search behavior in more detail. If such behavior is neither a metacognitive error nor a “passport effect,” then it must stem from a failure to inhibit looking. Measuring subjects’ inhibitory control and assessing whether it correlates with their propensity to make unnecessary looks in the tubes task may help identify the underlying mechanism.

Further research is required on the types of cues used in metacognition and which cognitive systems are accessible to metacognitive monitoring (see Hampton 2019). Significant progress has been made in the last 2 decades, but important gaps remain, with numerous questions concerning metacognition not only in NHPs, but other species too.

## Data Availability

Not applicable.
